# Supplementary material for: FERN – a Java framework for stochastic simulation and evaluation of reaction networks
Source: BMC Bioinformatics. 2008 Aug 29;9:356. doi: 10.1186/1471-2105-9-356 (PMC2553347; doi:10.1186/1471-2105-9-356)
Supplement: Additional file 1 — FERN distribution, Version 1.3. This archive contains the FERN source code and binaries as well as documentation and example models in FernML and SBML. [file 1471-2105-9-356-S1.zip › fern/doc/javadoc/fern/simulation/observer/MultiAmountIntervalObserver.html]

MultiAmountIntervalObserver


---


|  |  |  |
| --- | --- | --- |
| **Overview** \| **Package** \| **Class** \| **Use** \| **Tree** \| **Deprecated** \| **Index** \| **Help** | |  |
| **PREV CLASS**   **NEXT CLASS** | **FRAMES**    **NO FRAMES**     **All Classes** |  |
| SUMMARY: NESTED \| FIELD \| CONSTR \| METHOD | DETAIL: FIELD \| CONSTR \| METHOD |  |


---


## fern.simulation.observer Class MultiAmountIntervalObserver

```
java.lang.Object
  fern.simulation.observer.Observer
      fern.simulation.observer.IntervalObserver
          fern.simulation.observer.MultiAmountIntervalObserver
```

**All Implemented Interfaces:**: GnuPlotObserver

---

``` public class MultiAmountIntervalObserver extends IntervalObserver ```

Observes amounts of molecule species repeatedly after certain intervals. The amount of the
given molecules is summed up.

This observer does take repeats into account. If you repeat the simulation,
you will get an average over the results of each run.

**Author:**
:   Florian Erhard

---

**Constructor Summary**

`MultiAmountIntervalObserver(Simulator sim,
String presentationName,
double interval,
int... species)`
  
          Creates the observer for a given simulator, a given interval and given species indices

`MultiAmountIntervalObserver(Simulator sim,
String presentationName,
double interval,
String... speciesName)`
  
          Creates the observer for a given simulator, a given interval and given species names.


| **Method Summary** | |
| --- | --- |
| `protected  double` | `getEntityValue(int e)`             Gets the actual amount of a species. |
| `String[]` | `getStyles()`             Gets the styles for the columns. |

**Methods inherited from class fern.simulation.observer.IntervalObserver**

`activateReaction, finished, getFinalValue, getRecentData, isPlotQuality, isThetaMethod, setPlotQuality, setThetaMethod, started, step, theta, toGnuplot, toGnuplot, toGnuplotRecent, toGnuplotRecent, toString`

**Methods inherited from class fern.simulation.observer.Observer**

`applyLabelFormat, getLabel, getLabelFormat, getNumSimulations, getPrintWriter, getSimulator, getTheta, getTitlesCommand, print, setLabelFormat, setPrintWriter, setSimulator, setTheta`

**Methods inherited from class java.lang.Object**

`clone, equals, finalize, getClass, hashCode, notify, notifyAll, wait, wait, wait`

**Constructor Detail**

### MultiAmountIntervalObserver

```
public MultiAmountIntervalObserver(Simulator sim,
                                   String presentationName,
                                   double interval,
                                   int... species)
```

:   Creates the observer for a given simulator, a given interval and given species indices

    **Parameters:**: `sim` - simulator: `interval` - interval: `species` - species indices

---


### MultiAmountIntervalObserver

```
public MultiAmountIntervalObserver(Simulator sim,
                                   String presentationName,
                                   double interval,
                                   String... speciesName)
```

:   Creates the observer for a given simulator, a given interval and given species names.

    **Parameters:**: `sim` - simulator: `interval` - interval: `speciesName` - species names


**Method Detail**

### getEntityValue

```
protected double getEntityValue(int e)
```

:   Gets the actual amount of a species.

    :   **Specified by:**: `getEntityValue` in class `IntervalObserver`
    :   **Parameters:**: `e` - index of the entity **Returns:**: value of the entity

---


### getStyles

```
public String[] getStyles()
```

:   **Description copied from interface: `GnuPlotObserver`**
:   Gets the styles for the columns. If you don't want styles, just return null!

    :   **Returns:**: styles for the columns


---


|  |  |  |
| --- | --- | --- |
| **Overview** \| **Package** \| **Class** \| **Use** \| **Tree** \| **Deprecated** \| **Index** \| **Help** | |  |
| **PREV CLASS**   **NEXT CLASS** | **FRAMES**    **NO FRAMES**     **All Classes** |  |
| SUMMARY: NESTED \| FIELD \| CONSTR \| METHOD | DETAIL: FIELD \| CONSTR \| METHOD |  |


---
